# A case report on severe nivolumab-induced adverse events similar to primary sclerosing cholangitis refractory to immunosuppressive therapy

**DOI:** 10.1097/MD.0000000000025774

**Published:** 2021-06-11

**Authors:** Yuya Hirasawa, Kiyoshi Yoshimura, Hiroto Matsui, Yutaro Kubota, Hiroo Ishida, Jun Arai, Masashi Sakaki, Nao Oguro, Midori Shida, Makoto Taniguchi, Kazuyuki Hamada, Hirotsugu Ariizumi, Tomoyuki Ishiguro, Ryotaro Ohkuma, Takehiko Sambe, Atsushi Horiike, Chiyo K. Imamura, Eisuke Shiozawa, Satoshi Wada, Junji Tsurutani, Sanju Iwamoto, Naoki Uchida, Yuji Kiuchi, Genshu Tate, Shinichi Kobayashi, Takuya Tsunoda

**Affiliations:** a Division of Medical Oncology, Department of Medicine, Showa University School of Medicine; b Department of Clinical Immuno-Oncology, Clinical Research Institute of Clinical Pharmacology and Therapeutics, Showa University; c Division of Gastroenterology, Department of Medicine; d Division of Rheumatology, Department of Medicine; e Division of Clinical Pharmacology, Department of Pharmacology, Showa University School of Medicine; f Advanced Cancer Translational Research Institute, Showa University; g Department of Pathology and Laboratory Medicine, Showa University School of Medicine; h Department of Clinical Diagnostic Oncology, Clinical Research Institute for Clinical Pharmacology and Therapeutics, Showa University; i Division of Physiology and Pathology, Department of Pharmacology, Toxicology and Therapeutics, Showa University School of Pharmacy; j Division of Medical Pharmacology, Department of Pharmacology, Showa University School of Medicine; k Clinical Research Institute for Clinical Pharmacology and Therapeutics, Showa University, Tokyo, Japan.

**Keywords:** hepatitis, immune checkpoint inhibitor, immune-related adverse events, nivolumab, primary sclerosing cholangitis

## Abstract

**Introduction:**

Immune checkpoint inhibitors (ICIs), particularly anti-PD-1 antibody, have dramatically changed cancer treatment; however, fatal immune-related adverse events (irAEs) can develop. Here, we describe a severe case of sclerosing cholangitis-like irAE. We report the use of 3 immunosuppressive agents that resulted in the death of the patient due to treatment inefficacy. According to a postmarketing study of nivolumab, the frequency of ICI-related sclerosing cholangitis is 0.27% and that of ICI-related cholangitis is 0.20%. There have been 4 case reports of sclerosing cholangitis-like irAE, with imaging findings, including typical intrahepatic bile duct beaded constriction in primary sclerosing cholangitis. Treatment starts with prednisolone and is combined with an immunosuppressant in refractory cases. There are no reports of severe cases that ultimately led to death.

**Patients concerns:**

The patient is a 64-year-old male with Stage IV squamous cell lung carcinoma; he was hospitalized with abdominal pain and elevation of aspartate transaminase and alanine transaminase, approximately 4 months after ICI administration was suspended. This occurred because the patient treated with nivolumab as the second-line chemotherapy and developed type 1 diabetes mellitus after 11 courses.

**Diagnosis:**

A grade 3 increase in bilirubin was observed and he was diagnosed with sclerosing cholangitis, based on magnetic resonance cholangiopancreatography imaging and pathological findings of the liver and bile duct.

**Interventions:**

Prednisolone, mycophenolate mofetil, and tacrolimus combination therapy was administered.

**Outcomes:**

The treatment was difficult and failed. He died from liver failure 8 months after diagnosis. In this case, hepatitis and cholangitis, mainly alanine transaminase-dominant liver disorder, developed in the early stages of irAEs. Although he showed some improvement after prednisolone administration, bilirubin levels began rising again, and sclerosing cholangitis did not improve even with the use of 3 immunosuppressive agents recommended by the ESMO Clinical Practice Guidelines for immune-related hepatotoxicity management. Although the antitumor effect showed a complete response, liver failure led to death.

**Conclusion:**

This is the first case report on the ineffectiveness of triple immunosuppressant combination therapy recommended by the guidelines for immune-related hepatotoxicity. It is necessary to develop more appropriate treatment for severe sclerosing cholangitis-like irAE based on the robust evidence.

## 1 Introduction

Immune checkpoint inhibitors (ICIs) have dramatically changed cancer treatment but can sometimes cause life-threatening immune-related adverse events (irAEs). The frequency of ICI-related cholangitis and sclerosing cholangitis is very low.^[1]^ According to a postmarketing study of nivolumab from July 2014 to January 2020 in Japan, published on the website of Ono Pharmaceutical Co., Ltd., the frequency of ICI-related cholangitis is 0.27% (63/22,764 cases) and that of sclerosing cholangitis is 0.20% (46/22,764 cases).^[2]^ A protocol based on autoimmune hepatitis (AIH) is recommended for treating cholangitis and sclerosing cholangitis due to ICIs.^[3]^ In the recommended method, treatment for irAEs is initiated with prednisolone (PSL), mycophenolate mofetil (MMF), and tacrolimus (TAC), which was additionally administered for refractory cases. However, data on irAE cholangitis are lacking.^[4–10]^ In AIH, approximately 80% of patients with refractory PSL treatment improved following MMF supplementation; however, no studies have focused on irAE hepatitis and cholangitis.^[11,12]^ There are 2 case reports on hepatitis in which antithymocyte globulin was effective, but there is no data on cholangitis.^[13,14]^ In addition, the timing for using ursodeoxycholic acid (UDCA) has not been specified. Four case reports have described beaded stenosis of the intrahepatic bile ducts, which is typical of sclerosing cholangitis, 2 for nivolumab, and 2 for pembrolizumab.^[15–18]^ Most cases develop as abdominal pain or jaundice, and imaging tests show extrahepatic bile duct wall thickening and bile duct dilatation. In an advanced state, beaded stenosis of the peripheral bile duct is observed. Pathological findings include lymphocyte infiltration in the bile duct and hepatocyte necrosis on liver biopsy. Three of the 4 cases improved with treatment in these case reports, 1 improved with discontinuation of ICI, another improved with antibiotics and endoscopic treatment, and third improved with UDCA. One of the 4 cases did not improve and was refractory to UDCA, bezafibrate, and methylprednisolone (mPSL) 500 mg/d for 3 days followed by administration of 1.0 mg/kg/d PSL. We administered nivolumab as a second-line chemotherapy for squamous cell carcinoma of the lung after chemotherapy with carboplatin and nab-paclitaxel. This patient developed type 1 diabetes mellitus and ICI therapy was suspended, as the antitumor effect showed a complete response. Approximately 4 months later, the patient developed a condition resembling sclerosing cholangitis and was treated with PSL, MMF, and TAC but showed no improvement. He died of liver failure 8 months after diagnosis. This is the first case report of the ineffectiveness of triple immunosuppressant combined therapy recommended by the guidelines for irAEs similar to sclerosing cholangitis.

## 2 Case presentation

**Figure 1:**
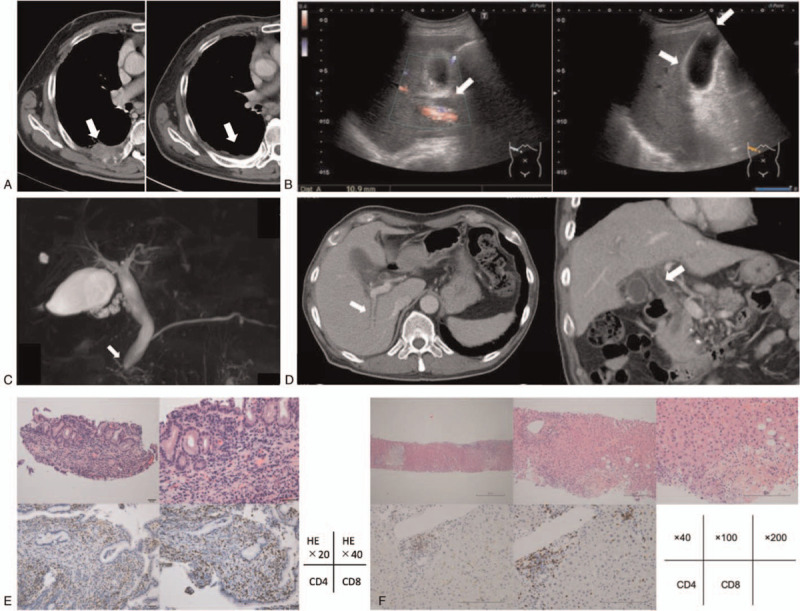
(A) Computed tomography (CT). Right pleural metastasis was observed before the start of nivolumab administration as the second-line chemotherapy (arrow). After 11 courses, the patient was diagnosed as diabetes mellitus, but the CT scan showed shrinkage of the metastasis. No abnormal accumulation was observed on PET-CT so that we determined complete response. (B) Abdominal ultrasound. Dilatation of the common bile duct was 10.9 mm (arrow). Mild wall thickening of the gallbladder (arrow), and cholelith less than 10 mm (arrow) were observed. (C) Magnetic resonance cholangiopancreatography (MRCP). Mild dilatation of the common bile duct was shown (arrow). Multiple low signal areas were observed, and common bile duct stones or debris was suspected. (D) CT at the second time. Periportal collar (arrow), approximately 11.0 mm of dilatation of the common bile duct, and bile duct wall thickening (arrow) were observed. (E) Pathological findings of bile duct biopsy. [Upper left] HE 20× [upper right] HE 40×, [Lower left] CD4 staining 200× [lower middle] CD8 staining 200×. There was no sign of malignant finding due to lack of atypical bile duct epithelial. Infiltration by lymphocyte and plasma cells was observed in the interstitium. CD4-positive T cells and CD8-positive T cells were also observed. IgG4 was negative. (F) Pathological findings of liver biopsy. [Upper left] HE 40× [upper middle] HE 100× [upper right] HE 200×, [Lower left] CD4 staining 200× [lower middle] CD8 staining 200×. Mild fibrous expansion of portal canal was observed, and small bile duct was normal. Only a few inflammatory cell infiltration was observed. CD8-positive T cells were found. Bile infarct was observed only in the lower right area of slide, and no inflammatory cells were found around necrosis. No bile plug was formed. There was no sign of malignant finding. PET = positron emission tomography.

**Figure 2:**
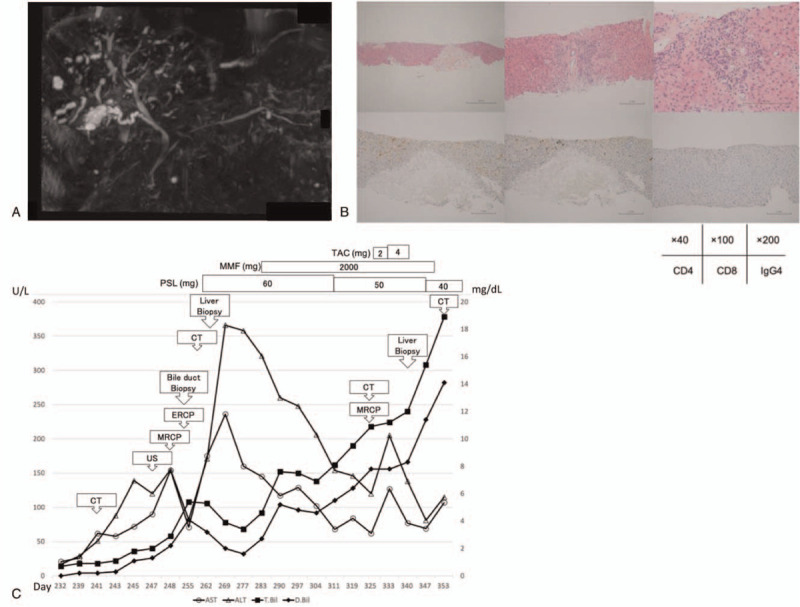
(A) MRCP when bilirubin level was re-increased at the second time. Periportal intensity was found on T2-weighted image. Beaded constriction and dilatation of the peripheral intrahepatic bile duct were detected. No dilatation of the common bile duct was found, but there was wall thickening of extrahepatic bile duct. There was also circumferential wall thickening of bladder. (B) Pathological findings of liver biopsy at the second time, [Upper left] HE 40× [upper middle] HE 100× [upper right] HE 200×, [Lower left] CD4 staining 200× [lower middle] CD8 staining 200× [lower right] IgG4 staining 200×. In periportal area, inflammatory cell infiltration was scarce, and small bile duct was normal. Biliary hyperplasia with cholestasis was observed. Hepatocyte necrosis was sporadic, and normal cells were sharply defined. It was punched out necrosis. There was no migration of lymphocytes at the site of necrosis and were only a few CD8-positive T cells around. IgG was negative. (C) Summary of clinical course and biochemical examination. MRCP = magnetic resonance cholangiopancreatography.

A 64-year-old man with Stage IV squamous cell lung carcinoma with right pleural metastasis was hospitalized on presenting with abdominal pain on the left side, increased aspartate transaminase (AST) and alanine transaminase (ALT) levels, and inflammation. Nivolumab (240 mg/body every 2 weeks) was administered for 11 courses as second-line chemotherapy after administration of carboplatin and paclitaxel as first-line of chemotherapy, discontinued because of the onset of type 1 diabetes mellitus. Right pleural metastasis was observed before administering nivolumab but shrinkage of the metastasis was observed at the onset of diabetes. As no abnormal accumulation was observed on positron emission tomography–computed tomography (CT), we determined complete response. Thereafter, administration of nivolumab was discontinued, and the patient was followed up (Fig. 1A). Four months after the last administration of nivolumab, he was hospitalized again, and grade 1 hepatic enzyme elevation was observed on admission (Table 1). CT showed mild periportal cuffing without dilatation of the common bile duct, whereas the results for hepatitis virus and autoantibodies were negative. Although antibacterial drugs were started, the liver enzyme and bilirubin levels continued increasing. Abdominal ultrasound (AUS) and ultrasound endoscopy revealed dilatation of the common bile duct, mild wall thickening of the gallbladder, and cholelith (Fig. 1B). Magnetic resonance cholangiopancreatography (MRCP) showed multiple low signal areas, suspected common bile duct stones or debris, and dilatation of the common bile duct (Fig. 1C). Endoscopic retrograde cholangiopancreatography (ERCP) revealed dilatation of the lower bile duct (11.6 mm) and a defect in the bile duct after contrast enhancement. Although it was cleaned by ERCP, no obvious stones or debris were discharged. Bile duct biopsy and bile cytology were also performed. Bilirubin continued to increase after ERCP, and liver enzymes reached Grade 3. CT showed bile duct wall thickening and intrahepatic periportal collar (Fig. 1D). The result of bile duct biopsy (on day 252) was infiltration of inflammatory cells CD4-positive and CD8-positive T cell into the stroma (Fig. 1E). There were no malignant findings. At this point, we diagnosed the patient with cholangitis and hepatitis as irAEs, and he was administered with 60 mg/d PSL (1.0 mg/kg/d). Two days later, liver biopsy was performed that revealed detachment with necrosis of hepatocytes and inflammatory cell infiltration of lymphocytes. The bile duct was normal, and no bile embolism was formed. Acute hepatitis with focal necrosis was diagnosed. Immunohistochemical staining revealed CD8- and CD4-positive T cells, with CD8-positive dominance and IgG staining negative for all cells (Fig. 1F). The level of hepatic enzymes and bilirubin decreased slowly; however, after 10 days, bilirubin levels increased again. Retesting of hepatitis virus and autoantibody showed negative results. The patient was diagnosed with exacerbation of hepatitis and cholangitis and was started on 2000 mg/d MMF; however, his bilirubin levels worsened. The dose of PSL was reduced to 50 mg/d because of its poor efficacy and development of cytomegalovirus antigenemia. Later, the increase in bilirubin worsened to Grade 4 (total bilirubin 10.9 mg/dL, direct bilirubin 7.8 mg/dL). MRCP was performed for the second time, and beaded constriction and dilatation of the peripheral intrahepatic bile duct were detected (Fig. 2A). Considering ICI-induced sclerosing cholangitis, 2 mg/d TAC was initiated, and the blood concentration target was controlled at 6.0 to 10.0 ng/mL, whereas TAC was increased to 4 mg/d. However, the bilirubin level did not decrease. The patient was discontinued from TAC because of its poor efficacy and complication of infections, such as bacterial, fungal, and cytomegalovirus infections. A liver biopsy was performed for the second time, and pathological findings suggested that inflammatory cell infiltration of the liver parenchyma was scarce. Hepatocyte necrosis or dropout was observed along with mild biliary hyperplasia of the Gleason sheath (Fig. 2B). Based on these results, the patient was diagnosed with large bile duct obstruction with bile infarction. Later, because of the poor therapeutic effect and infection control, MMF was discontinued and PSL administration was gradually reduced. Liver transplantation was also considered; however, it was not indicated because the recurrence-free period was too short. Although Grade 4 bilirubin persisted, the liver enzyme elevation improved to Grade 1–2 (Fig. 2C). The patient was discharged after treatment for the infection. The patient died from liver failure at 8 months after the diagnosis of irAEs.

**Table 1:** Clinical findings upon admission.

Vital signs								
BT	36.9	°C	BP	117/79	Mm Hg			
RR	20	/min	SpO2	98	% (room air)	HR	**103**	/min
Labo Data								
WBC	**11900**	/μL	PT-INR	1.09		AST	**62**	U/L
Neut	**88.7**	%	APTT	48.7	s	ALT	**51**	U/L
Lymp	6.0	%	D-dimer	0.95	μg/mL	ALP	308	U/L
RBC	490	×10^4^/μL	Fib	596	mg/dL	γGTP	58	U/L
Hb	14.4	g/dL	TP	7.0	g/dL	LDH	**231**	U/L
Hct	42.2	%	Alb	3.4	g/dL	BUN	25.1	mg/dL
Plt	33.6	×10^4^/μL	T.Bil	0.9	mg/dL	Cre	0.82	mg/dL
PT	83	%	D.Bil	0.2	mg/dL	CRP	**14.62**	mg/dL

γGTP = gamma-glutamyl transpeptidase, Alb = albumin, ALT = alanine aminotransferase, APTT = activated partial thromboplastin time, AST = asparate aminotransferase, BP = blood pressure, BT = body temperature, BUN = blood urea nitrogen, Cre = creatinine, CRP = C-reactive protein, D-Bil = direct bilirubins, Fib = fibrinogen quantity, Hb = hemoglobin, Hct = hematocrit, HR = heart rate, LDH = lactate dehydrogenase, Lymp = lymphocyte, Neut = neutrophil, Plt = platelet count, PT = prothrombin time, PT-INR = prothrombin time-international normalized ratio, RBC = red blood cell count, RR = respiratory rate, SpO2 = arterial oxygen saturation of pulse oxymetry, T.Bil = total bilirubins, TP = total protein, WBC = white blood cell count.

## 3 Discussion and conclusions

We administered nivolumab for squamous cell carcinoma of the lung. Thereafter, type 1 diabetes mellitus developed, and ICI administration was discontinued. About 4 months after the last administration, the patient experienced an irAE that resembled primary sclerosing cholangitis with abdominal pain as the first symptom and was treated with PSL, MMF, and TAC; however, this combination therapy was ineffective.^[19–21]^ Our case was compared with similar cases from other reports describing beaded constriction of the intrahepatic bile duct based on imaging findings (Table 2).

**Table 2:** Clinical and pathological characteristics of our case and similar cases reported by multiple facilities.

First author	Age Sex	Primary disease Drugs Cycle	Symptoms/ timing of onset	Imaging findings of bile ducts (1)Dilatation (2)Thickening wall (3)Irregular Narrowing	Pathological findings (1) CD8+ T cell infiltration	Treatment	Improve
Noda^[15]^	57 F	NSCLC(Ad) Nivolumab 7 cycles	Fever Abdominal pain/7 mo after stopping ICI	[AUS] (1)+ (2)+ [CT] (1)+ (2)+ (3)+ [MRCP] (1)+ (3)+ [EUS] (1)+ (2)+ [ERCP] (1)+ (2)+	N/A	UDCA	+
Kono^[16]^	69 F	GC Nivolumab 2 cycles	Jaundice/2 mo after stopping ICI	[AUS] (1)+ [CT] (1)+ [MRCP] (1)+ [EUS] (1)+ [ERCP] (1)+ (3)+	N/A	Antibiotic Therapy Endoscopic Intervention	+
Ogawa^[17]^	73 M	Melanoma Pembrolizumab 7 cycles	None/continuing ICI	[CT] (1)+ (2)+ [EUS] (1)+ (2)+ (3)+ [ERCP] (1)+ (2)+ (3)+	[Bile duct] (1)+ Destruction with fibrosis	Discontinue ICI	+
Koya^[18]^	66 F	SCLC Pembrolizumab 5 cycles	Epigastric pain/continuing ICI	[AUS] (1)+ [CT] (1)+ (2)+ [MRCP] (1)+ (3)+ [EUS] (1)+ (2)+ [ERCP] (1)+ (3)+	[Liver/ portal area] (1)+ Bile ductular proliferation Cholestatic changes Canalicular bile plugs [Bile duct] (1)+	UDCA Bezafibrate mPSL to PSL	−
Our case	64 M	NSCLC(Sq) Nivolumab 11 cycles	Left abdominal pain/4 mo after stopping ICI	[AUS] (1)+ [CT] (1)+ (3)+ [MRCP] 1st: (1)+ 2nd: (1)+ (3)+ [EUS] (2)+ [ERCP] (1)+	[Liver] (1)+ Bile ductular proliferation Cholestatic changes [Bile duct] (1)+	UDCA PSL MMF TAC	−

Ad = adenocarcinoma, AUS = abdominal ultrasound, CT = computed tomography, ERCP = endoscopic retrograde cholangiopancreatography, EUS = endoscopic ultrasonography, GC = gastric cancer, ICI = immune checkpoint inhibitor, MMF = mycophenolate mofetil, mPSL = methylprednisolone, MRCP = magnetic resonance cholangiopancreatography, NSCLC = non-small cell lung cancer, PSL = prednisolone, SCLC = small cell lung cancer, Sq = squamous cell carcinoma, TAC = tacrolimus, UDCA = ursodeoxycholic acid.

The frequency of ICI administration is 2 to 7 courses, whereas it was 11 courses for this case.^[15–18]^ At onset, 2 patients were being administered the treatment, and 2 patients experienced onset after treatment discontinuation. The period from discontinuation to onset was around 7 months in the most delayed onset case and 4 months in the present case.^[15]^

The first symptoms were mostly abdominal pain and jaundice, also observed in our case.^[15,16,18]^ In the early stage of onset, AUS and ERCP tend to show dilatation of the common bile duct and increased wall thickness of the bile duct and gall bladder. Later, CT and MRCP show beaded constriction and dilation of the peripheral intrahepatic bile duct.^[15–18]^ This case also followed the same course but the degree of bile duct stenosis was high and treatment response was poor. The stenosis pattern in this case was similar to that of typical primary sclerosing cholangitis. Some mild cases even present with typical imaging findings of primary sclerosing cholangitis on an initial imaging test; therefore, it is unclear whether severe cholangitis became sclerosing cholangitis pathologically.^[16,17]^ In addition, AUS may be useful for measuring qualitative changes over time in the bile ducts and assessing the progress of the condition noninvasively.

Pathological findings typically include nonspecific inflammatory cell infiltration into the bile duct or CD4-positive/CD8-positive T cell infiltration.^[17,18]^ The liver showed portal hyperplasia of the bile duct, hepatocellular necrosis, mild inflammatory cell infiltration, and absence of IgG4.^[18]^ Although similar findings were observed in this case, hepatic biopsy at the time of exacerbation of sclerosing cholangitis confirmed the loss of necrosis, and the inflammation findings disappeared. The major condition in this case was hepatic enzyme and bilirubin elevation because of hepatocyte necrosis based on bile duct obstruction. Regarding hepatic enzyme elevation, the possibility that hepatitis was complicated in the early stage of onset cannot be ruled out.^[22–25]^ In 3 case reports, AST and ALT were elevated, and hepatic enzyme elevation occurred even with cholangitis alone.^[16–18]^ Gamma-glutamyl transferase, alkaline phosphatase, and bilirubin levels are also elevated in hepatitis, and thus are not specific to cholangitis and cannot be used to predict severe cases.^[13,14]^

Currently, treatment is recommended according to the AIH algorithm, which has been reported to improve 3 cases: 1 was treated with UDCA, another with antibacterial drugs and endoscopic treatment, and the third with discontinuation of ICIs.^[15–17]^ The other case was reported for refractory case treated with 900 mg/d UDCA, 400 mg/d bezafibrate, and 500 mg/d mPSL for 3 days followed by 1.0 mg/kg/d PSL administration.^[18]^ In our case, the 3 drugs were used in accordance with AIH guidelines but failed to improve the condition. No similar results have been reported previously. A treatment protocol for sclerosing cholangitis has not been established, and the protocol is based on AST and ALT for hepatitis.^[3]^ The validity of this approach, appropriate drug selection, and doses are unknown.

Hepatitis may have overlapped with other conditions in this case, as AST and ALT levels increased markedly with hepatocyte necrosis of bile infarction. As elevations in AST and ALT levels reduced, the pathological condition seemed to improve. However, if primary sclerosing cholangitis was the primary condition, additional treatment with immunosuppressants could have been considered earlier based on bilirubin levels if AST and ALT were decreasing. Moreover, there is no clear standard on how to use UDCA. For PSL, it is difficult to determine whether a 1.0 or 2.0 mg/kg should be used. In our case, 2.0 mg/kg/d was acceptable, but 1.0 mg/kg/d has reportedly been used in many cases.^[7,8,18,25]^ The most appropriate use of mPSL pulse therapy and whether the second appropriate agent may be MMF based on therapy for AIH are also unclear.

Here, we report a case of irAE with a variable history followed by the development of sclerosing cholangitis on ICI treatment. We compared this case with similar cases reported by multiple facilities.^[15–18]^ In our case, combined therapy of 3 immunosuppressive agents was used but the patient died. In the case of irAE with sclerosing cholangitis among hepatotoxic cases, intensive treatment based on evaluation of bilirubin levels rather than AST and ALT levels may be important. In addition, AUS is useful for assessing deteriorated and severe cases earlier. Studies are needed to collect additional data on biliary injury cases and establish a suitable treatment.

## Acknowledgments

The authors would like to thank Editage (www.editage.com) for English language editing. The authors also thank Manami Kobayashi for the advice on article composition.

## Author contributions

**Conceptualization:** Yutaro Kubota.

**Writing – original draft:** Yuya Hirasawa.

**Writing – review & editing:** Kiyoshi Yoshimura, Hiroto Matsui, Yutaro Kubota, Hiroo Ishida, Jun Arai, Masashi Sakaki, Nao Oguro, Midori Shida, Makoto Taniguchi, Kazuyuki Hamada, Hirotsugu Ariizumi, Tomoyuki Ishiguro, Ryotaro Ohkuma, Takehiko Sambe, Atsushi Horiike, Chiyo K. Imamura, Eisuke Shiozawa, Satoshi Wada, Junji Tsurutani, Sanju Iwamoto, Naoki Uchida, Yuji Kiuchi, Genshu Tate, Shinichi Kobayashi, Takuya Tsunoda.
